# Proximal and distal control for ligand binding in neuroglobin: role of the CD loop and evidence for His64 gating

**DOI:** 10.1038/s41598-019-41780-3

**Published:** 2019-03-29

**Authors:** Cécile Exertier, Lisa Milazzo, Ida Freda, Linda Celeste Montemiglio, Antonella Scaglione, Gabriele Cerutti, Giacomo Parisi, Massimiliano Anselmi, Giulietta Smulevich, Carmelinda Savino, Beatrice Vallone

**Affiliations:** 1 Dip. di Scienze Biochimiche “A. Rossi Fanelli”, Sapienza Università di Roma, P.le A. Moro 5, 00185 Rome, Italy; 2 Istituto Pasteur-Fondazione Cenci Bolognetti, Dip. di Scienze Biochimiche “A. Rossi Fanelli”, Sapienza Università di Roma, P.le A. Moro 5, 00185 Rome, Italy; 3 Dip. di Chimica “Ugo Schiff”, Università di Firenze, Via delle Lastruccia 3–13, 50019 Sesto Fiorentino (FI), Italy; 4 Institute for Microbiology and Genetics, Georg-August University Göttingen, Justus-von-Liebig-Weg 11, 37077 Göttingen, Germany; 5 CNR Institute of Molecular Biology and Pathology, P.le A. Moro 5, 00185 Rome, Italy; 6 Present Address: Neuroscience Initiative, CUNY Advanced Science Research Center, 85 St. Nicholas Terrace, 10031 New York, USA; 7 Present Address: Department of Physiology and Cellular Biophysics, Russ Berrie Pavilion, Columbia University Medical Center, 1150 St Nicholas Ave, 10032 New York, USA

## Abstract

Neuroglobin (Ngb) is predominantly expressed in neurons of the central and peripheral nervous systems and it clearly seems to be involved in neuroprotection. Engineering Ngb to observe structural and dynamic alterations associated with perturbation in ligand binding might reveal important structural determinants, and could shed light on key features related to its mechanism of action. Our results highlight the relevance of the CD loop and of Phe106 as distal and proximal controls involved in ligand binding in murine neuroglobin. We observed the effects of individual and combined mutations of the CD loop and Phe106 that conferred to Ngb higher CO binding velocities, which we correlate with the following structural observations: the mutant F106A shows, upon CO binding, a reduced heme sliding hindrance, with the heme present in a peculiar double conformation, whereas in the CD loop mutant “Gly-loop”, the original network of interactions between the loop and the heme was abolished, enhancing binding via facilitated gating out of the distal His64. Finally, the double mutant, combining both mutations, showed a synergistic effect on CO binding rates. Resonance Raman spectroscopy and MD simulations support our findings on structural dynamics and heme interactions in wild type and mutated Ngbs.

## Introduction

Phylogenetically ancient globins such as neuroglobin (Ngb), globin X and androglobin are endowed with heme iron hexacoordination, whereas pentacoordinated myoglobins (Mb) and hemoglobins (Hb), characterized by a complex respiratory role, appeared later in evolution. The current hypothesis is that hexacoordination could be the oldest coordination scheme. Furthermore, this iron binding mode triggers a protein conformational reorganization upon ligand binding that could play a role in gas sensing^1^.

Regardless of the variety of functions that globins may perform (NO metabolism, lipid metabolism, anti-apoptosis, signal transduction, detoxification of ROS/RNS, respiration, O_2_ diffusion), they share a common fold, with 8 helices named from A to H, the so-called 3-on-3 *α*-helical sandwich^2^, and some very specific structural elements. The sequence conservation of globin structure involves two universally invariant positions: a histidine at the 8th position of the F-helix, that binds the heme iron proximally, and a phenylalanine in position CD1 that is packed against the heme^3^. Sequence alignment of a large set of globins also highlighted the strong conservation of other residues such as a histidine in position E7 on the heme distal side, which, in some cases, is involved in heme hexacoordination and/or in the stabilization of bound ligands. Sequence conservation in globins highlights residues involved in heme packing (His(F8), Phe(CD1), Leu(B10), Phe(B14), Phe(CD4)) or participating to the early stage of globin folding, with residues A8, G16, G12, A12, H5, H12 forming the A, G and H helices which act as a folding nucleus^4^.

Ngb is over-expressed in hypoxic conditions and it may be involved in gas/redox sensing, radical scavenging, and/or signal transduction^5^. However, its precise mechanism of action remains to be clarified, despite Ngb function involves binding of diatomic gases, such as O_2_, NO and CO. Notably, hexacoordination in Ngb accounts for a peculiar structural rearrangement and complex kinetics upon ligand binding^6–13^, which takes place when the distal histidine (His(E7)64), engaged in internal iron hexacoordination, dissociates from the heme. The dissociation of His64 is the rate-limiting step for ligand binding and it triggers a repositioning of the heme which slides further inside a large internal cavity. This movement is associated with the motion of helices and loops^14–16^, and probably confers to Ngb a more stable conformation. More recently, molecular dynamics (MD) simulations supported a His64 gating out movement coupled with a motion of the CD corner upon ligand binding, larger than the one described by crystallography^17,18^. Another structural feature of neuroglobin is the double insertion of the heme with 70:30 proportion^14^.

Artificial point mutants of Ngb have been utilized to pinpoint major structural determinants for ligand binding regulation. Pentacoordinated Ngbs obtained by substituting the distal His64 with Leu, Val or Gln, display fast ligand binding and low heme iron auto-oxidation^6,11,19^. Proximal heme pocket mutants (M144W, and F106W) showed a hampered heme sliding, affecting both binding velocity and affinity^20^. Tyr44, in the CD loop, is an important heme-interacting residue involved in regulating binding kinetics^21^ since its mutation into an aspartate yielded a 15-fold increase in O_2_ binding velocity and a 1.5-fold increase in O_2_ affinity. Finally, the CD loop was shown to be involved in the fine tuning of iron coordination by swapping CD corners of human Ngb and sperm whale Mb (swMb), which led to gain of hexa-coordination in swMb^22^.

In this study, we spectroscopically and structurally characterized three Ngb mutants aiming at pinpointing key features: a CD loop mutant with enhanced flexibility (Gly-loop), the F106A mutant and the double Gly-loop/F106A mutant (Supplementary Fig. S1). We probed the role of heme sliding in the internal cavity, facilitated by reducing the bulk in position 106 and the role of the CD loop in governing ligand affinity and hexacoordination.

## Results and Discussion

### CO binding at 25 °C by rapid mixing

**Figure 1:**
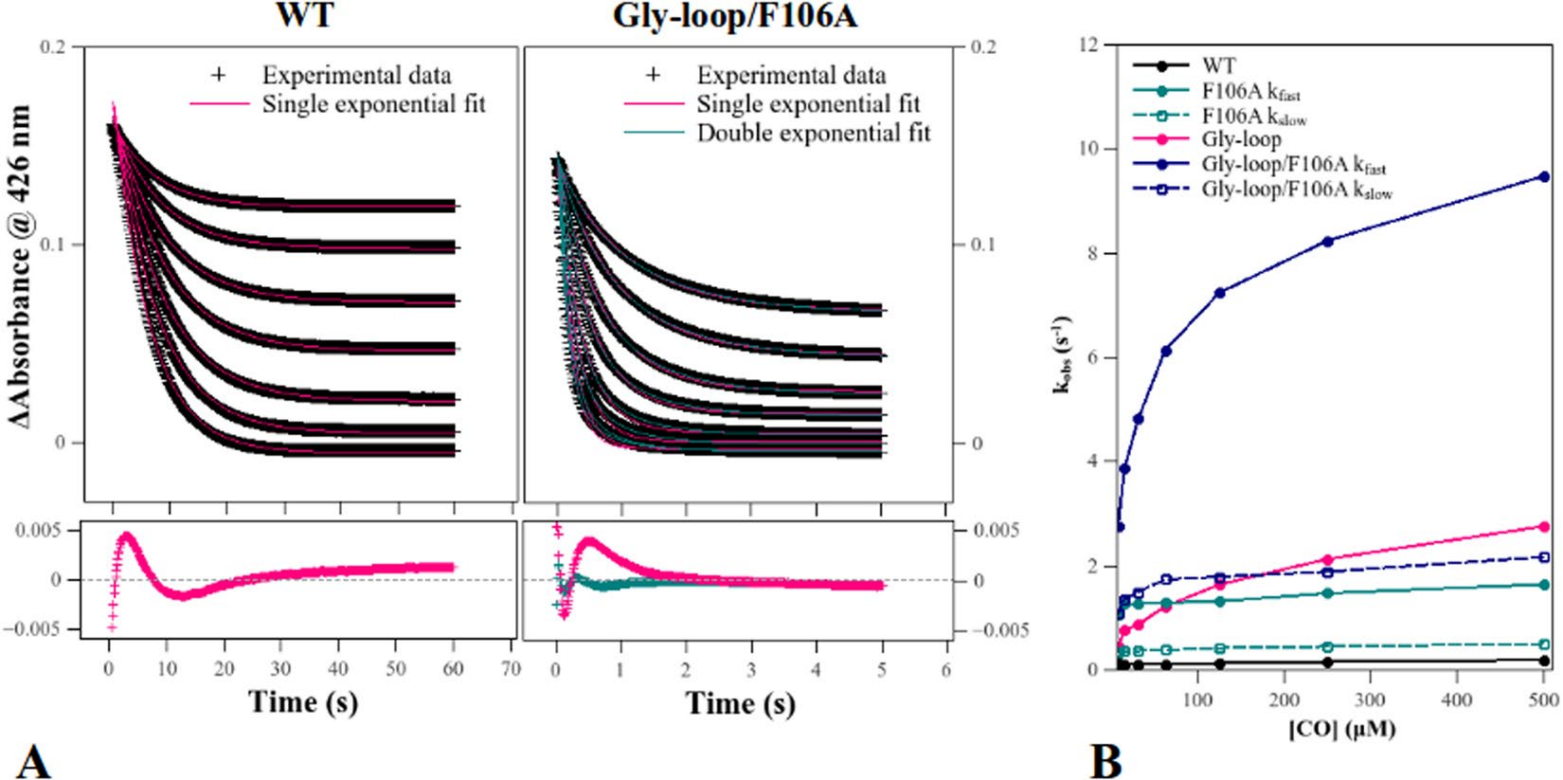
CO binding to ferrous neuroglobin mutants at 25 °C. CO binding rate constants k_*obs*_ were extracted from fitting experimental kinetic traces as single or double exponentials (**A**) and plotted as a function of CO concentration (**B**).

Wild type (WT) and mutant Ngbs were mixed with CO at 25 °C using a stopped flow apparatus. Traces were fitted as single (WT and Gly-loop, Fig. 1A) or double exponentials (F106A and Gly-loop/F106A, Fig. 1A). As observed previously^20^, CO binding to WT Ngb exhibits a mono-phasic decay at 426 nm, and so does the Gly-loop mutant. However, for Phe106 mutants (F106A and Gly-loop/F106A), a double exponential trend was observed, suggesting the existence of two populations. Both Gly-loop and F106A mutations significantly increase CO binding velocities. The Gly-loop mutation has the largest effect on ligand association rates (k_*obs*_(WT) = 0.19 s^−1^ vs k_*obs*_ (Gly-loop) = 2.8 s^−1^ at 500 *μ*M CO) compared to the F106A mutation (k_*obs*_ slow(F106A) = 0.51 s^−1^ and k_*obs*_ fast(F106A) = 1.7 s^−1^, Fig. 1B). However, these mutations have a synergistic effect rather than an additive one, as shown by the increase of rate constants (k_*obs*_ slow = 2.2 s^−1^ and k_*obs*_ fast = 9.5 s^−1^) for the double Gly-loop/F106A mutant. Unlike WT and F106A Ngbs, both mutants containing the CD loop mutation (Gly-loop and Gly-loop/F106A) show a strong dependence on CO concentration in their binding rate constants k_*obs*_.

Notably, CO binding to hexacoordinated Ngb is scarcely dependent on ligand concentration^20,21^, since the main factor affecting binding is the spontaneous dissociation of the distal histidine (His64). This suggests that, in the Gly-loop mutant, the rupture of the His64–heme iron bond is still the rate limiting step, but an increase in its velocity (or decrease in the bond re-formation velocity) causes a marked dependence on CO concentration. For the F106A mutant the fraction of the amplitude of the two phases at 500 *μ*M CO is 64% for the fast one and 36% for the slow one, for the Gly-loop/F106A, we observed that the slow fraction is of a lesser extent (13%, Supplementary Table S2).

For all the mutants, the increase in binding velocities is coupled to an overall augmented CO affinity with respect to WT, as calculated from the saturation at different CO concentrations. (Supplementary Fig. S2 and Table S2).

### Structural Characterization of neuroglobin mutants by Resonance Raman (RR)

An increased velocity of the Gly-loop/F106A mutant in CO binding might be a consequence of the presence of some pentacoordinated species due to the modification of the CD loop^22^. However, resonance Raman spectroscopy did not reveal any pentacoordinated species in the ferrous form or in the photolyzed CO sample (Supplementary Fig. S3). This result is consistent with the H64L mutant behaviour (bimolecular CO rebinding rates or Ngb H64L = 230 *μ*M.s^−1^ at 20 °C^19^), which is much faster than the fast phase detected for Gly-loop/F106A.

**Figure 2:**
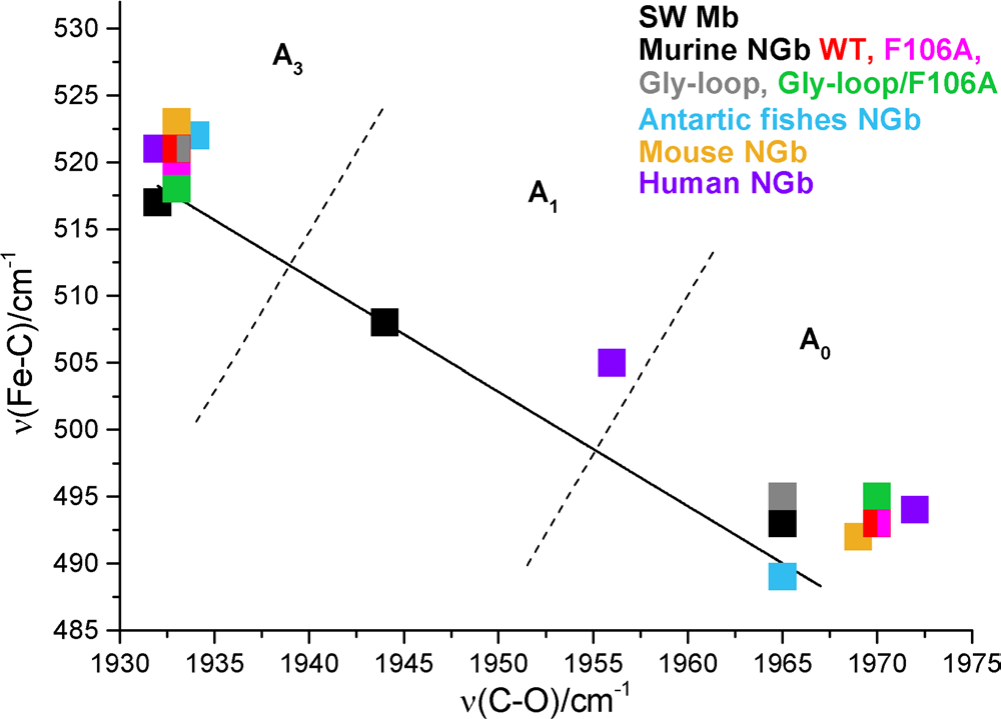
Back-bonding correlation line of the *ν*(Fe-C) and *ν*(C-O) stretching frequencies of various Ngbs^23–25,35^. The corresponding data of sperm whale Mb are also reported^36,37^. The dotted lines indicate the approximate delineation between the frequency zones of the A_0_, A_1_, and A_3_ forms. The human Ngb and swMb show also a third weak H-bonded conformer (A_1_) at 505/1956 and 508/1946 cm^−1^, respectively, not reported in the Table S3.

The RR spectra of the CO complexes of the WT, the F106A, and double Gly-Loop/F106A mutant (Supplementary Fig. S4) are very similar and show, consistently with other Ngbs^23–25^, the presence of two protein conformers: an open A_0_ form [*ν*(Fe-C) at 496 cm^−1^ and *ν*(CO) at 1970 cm^−1^] and a more abundant closed one, A_3_ [*ν*(Fe-C) at 521 (WT) and 518 (mutants) cm^−1^, and *ν*(CO) at 1933 cm^−1^], whose CO is H-bonded with His64. These data are reported in Fig. 2 which shows the back-bonding correlation line of the ν(Fe-C) and ν(CO) stretching frequencies for various Ngbs and swMb (the frequencies are reported in Supplementary Table S3).

It can be seen that, after CO binding, His64 can adopt two conformations, one that strongly interacts with the CO (closed form) and one with His64 far from the ligand (open cavity, consistent with a swung out His64). The Gly-loop mutant behaves differently, being the A_0_ bands quite intense. We tentatively assigned this band to two overlapped A_0_ open conformations.

### CO binding kinetics of Ngb F106A is consistent with a double heme conformation

Crystal structures of unliganded hexacoordinated F106A (1.8 Å) and carbomonoxy-F106A (F106A-CO, 1.75 Å), were determined to observe the effect of removal of the steric barrier to heme sliding posed by Phe106.

At this resolution, we clearly detect the double heme conformation (Supplementary Fig. S5A and S5B): the B conformer (canonical, 30% in ferric F106A, 27% in F106A-CO) and the A one (reversed, 70% in ferric F106A, 73% in F106A-CO). The assignment of the relative population of the two heme insertion modes is based on refinement and electron density residuals. The estimated error in this determination is therefore about 3%. As compared to WT Ngb, the structural difference between the two conformers is larger, likely due to the wider heme cavity, created by the Phe to Ala substitution that allows conformer B to adopt a deeper position within the pocket. We also observed double conformations for proximal and distal histidines.

The geometry of the His64-Fe-His96 bond, for both conformers, appears to be different with respect to WT Ngb (Supplementary Table S4) and it might contribute to reactivity with ligands. However, the most striking observation about heme conformations is the relative position of the A and B conformers, that are laterally and vertically shifted (Supplementary Fig. S5), with their iron atoms being about 1.0 Å and 0.90 Å apart, respectively in the unliganded and in the carbomonoxy form. In addition, the Fe-Fe distance between hexacoordinated F106A and F106A-CO shows, upon CO binding, a displacement of the heme A conformer of about 2.3 Å and of 2.9 Å for the heme B conformer, larger than the one observed for WT Ngb (2.0 Å^15^).

The enhanced displacement of B conformer is due to the absence of Phe106 ring onto which the heme leans in the WT protein. Consistently, Phe(CD1)42, a conserved residue involved in the heme packing through *π*-*π* stacking interactions, adopts a double conformation in a 70:30 proportion (lateral displacement of 1.0 Å at the level of Phe42 C*ζ*, not shown) in the structure of unliganded F106A.

In the F106A-CO structure, the absence of Phe in position 106, associated with the two heme insertion modes, induces residues such as Trp148 and the whole FG loop to explore multiple conformations upon heme sliding (Supplementary Fig. S6A–C). This structural feature could account for the biphasic behaviour detected in CO binding. The heme double conformation has already been proposed to be responsible for the slightly biphasic ligand binding in human Ngb, absent in murine WT Ngb, and which is greatly enhanced in murine F106A Ngb, due to the enhanced difference of A and B heme conformer positions^26,27^.

**Figure 3:**
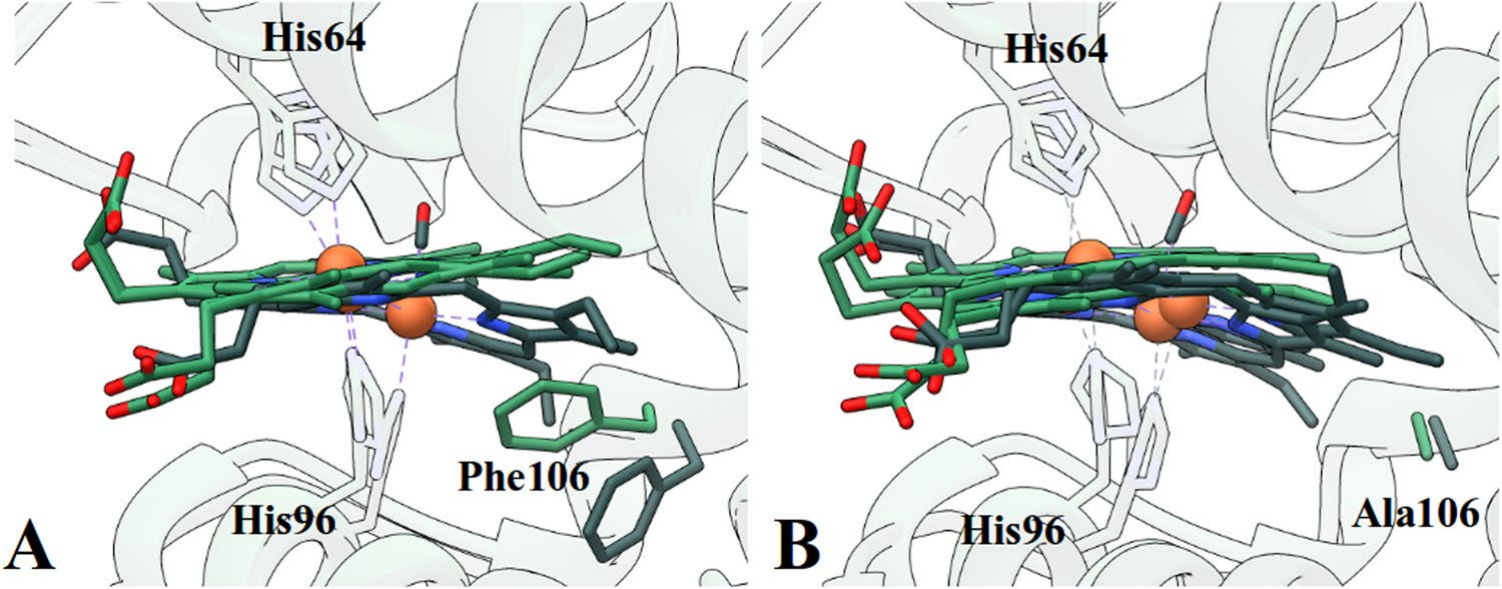
Comparison of wild type and F106A neuroglobin structures. (**A**) Unbound (green) and carbo-monoxy (grey) WT Ngb structures^14,15^. (**B**) Unbound (green) and carbo-monoxy (grey) F106A Ngb structures.

Finally, the absence of the aromatic side chain of Phe106 accounts for faster CO binding in both heme conformers, considering that in the WT, the phenylalanine ring has to flip to allow the heme to slide inside the cavity^15,20^ (Fig. 3A), indeed, in the F106A mutant, the alanine side chain stays put upon heme repositioning (Fig. 3B). In mutant F106W^20^, where steric hindrance is increased, CO binding velocity decreases of about five times and affinity is lowered by an order of magnitude (Supplementary Table S2).

The evaluation of the fast and slow binding phases in CO mixing experiment allows us to attribute to the B conformer the slow binding fraction and to the A conformer the fast binding one. An additional feature contributing to the increased CO binding velocities might reside in enhanced acceleration of the spontaneous rupture of the His64-Fe bond due to variations in its geometry reported in Supplementary Table S4.

### The disruption of the heme-CD loop interaction network is responsible for increased CO binding velocities in the Gly-loop mutant

**Figure 4:**
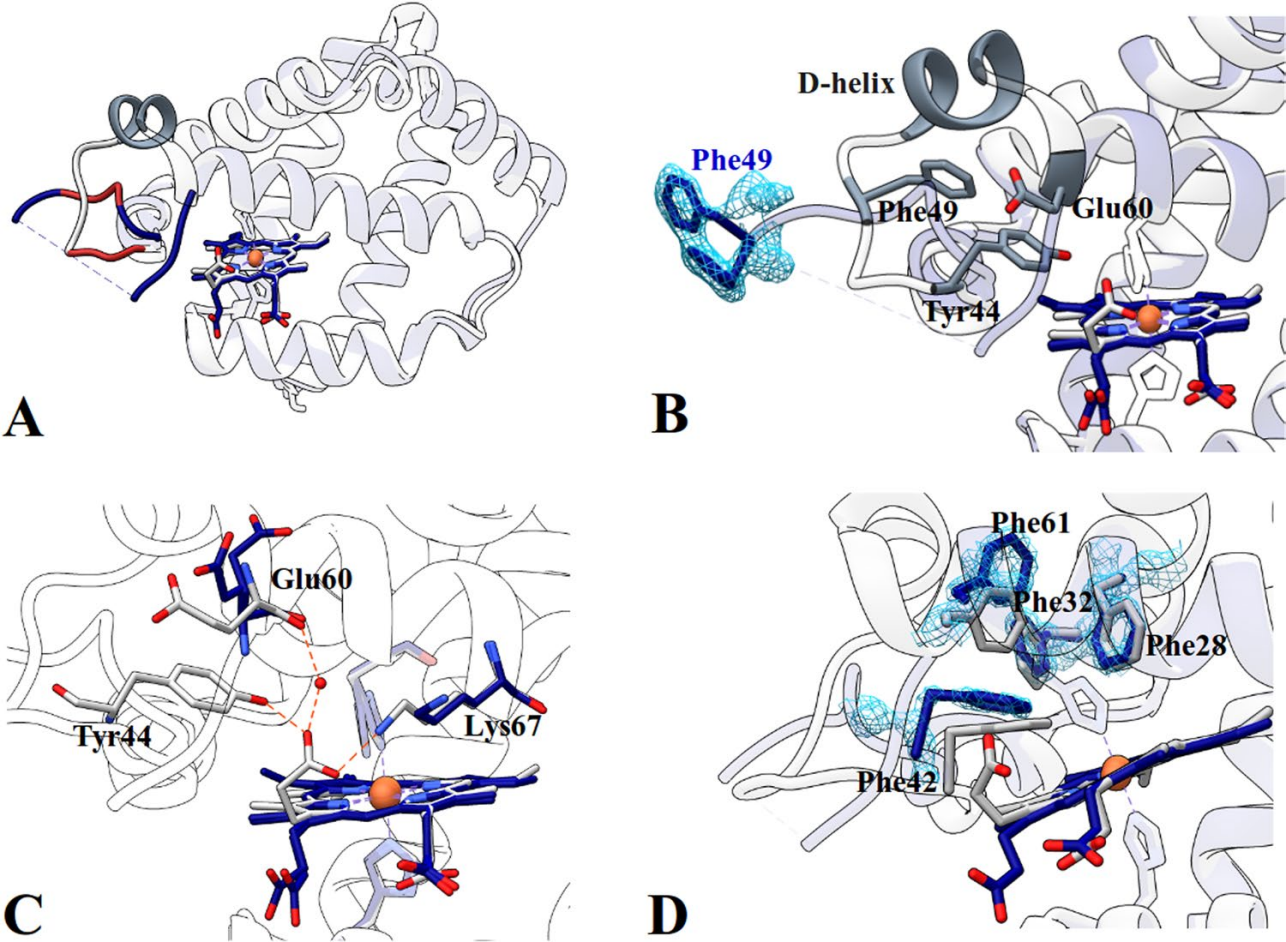
Superposition of Ngb Gly-loop (blue) and Ngb WT (grey) structures. (**A**) Comparison between the overal fold of the WT and the Gly-loop mutant. The segment missing from electron density maps (Phe49-Ser57) is indicated by a dashed line, the mutated sequence is in red, and the folded D-helix is indicated in dark grey. (**B**) In WT, the D-helix leans on Phe49, for the Gly-loop mutant, the 2Fo-Fc map is contoured at 1*σ* for Phe49. (**C**) Network of interactions between Tyr44, Glu60, Lys67, one of the heme propionates and a water molecule in WT Ngb. In the Gly-loop mutant, this network is abolished due to the absence of Tyr44. (**D**) The canonical hydrophobic patch in neuroglobin involved in heme support and maintenance of the globin fold is perturbated.

The global Ngb 3D architecture is conserved in the CD corner “Gly-loop” mutant as shown in Fig. 4A. The double conformation of the heme in 70:30 proportion, along with corresponding histidine double conformations, is present and His64(N*ε*)-Fe-His96(N*ε*) angles are unchanged with respect to WT (Supplementary Table S4). Besides some alternate conformations of the N- and C-termini, of the AB and EF loops, due to crystal packing changes, only the CD loop was significantly altered. The lack of well defined electronic density did not allow to reconstruct the last three residues (aa 50–52) of the CD loop Fig. 4A, grey dashed line), which was completely reshaped. Moreover the D-helix was disordered (Fig. 4A, red) and no electronic density was observed up to the beginning of the E-helix at Pro59 (Fig. 4A,B).

Figure 4C shows the alteration of intra-molecular interactions in the Gly-loop Ngb. In the WT protein, the oxygen of one of the propionates interacts with Lys67 (Fig. 4B), this propionate and Tyr44 constitute a barrier to the swinging out of His64 (Supplementary Fig. S7) which in MD simulations, can take place only upon the upward movement of the CD loop and propionate flipping^17^. Tyr44 and Lys67 firmly interact at a 2.5 Å distance with a water molecule bridging the propionate to the main chain oxygen of Glu60. Owing to the mutation of Tyr44 into a glycine, the propionate flips toward the proximal side of the heme, and the amino group of Lys67 is rotated by 180°. Moreover in WT Ngb, the bond between Tyr44 and the heme propionate favors a conformation of the CD loop in which the D-helix leans on the Phe49 aromatic ring (Fig. 4B). The absence of Tyr44, that is not pinning any more the loop to the heme, allows the displacement of Phe49 which might explain the D-helix destabilization. In addition to pinning the CD loop to the heme propionate, Tyr44 appears to be part of the aromatic core around which the CD-loop-D helix segment is wound, with the significant stacking of the aliphatic portion of Glu60 onto its phenyl moiety. The CD-loop-D-helix unit seems therefore to be an intrinsically dynamic segment, whereas the C- and E-helices appear to constitute a rigid scaffolding in Ngb. The mobilization of the CD-loop-D-helix unit could facilitate the His64 dissociation from the heme iron, unlocking its swing-out displacement, thus leading to the increased CO binding velocities observed by rapid mixing. Indeed, faster O_2_ association was also observed for Tyr44Asp Ngb, which has a behaviour remarkably similar to the Gly-loop mutant, including enhanced ligand concentration dependence and ligand affinity (P_50_(Y44D) = 1.3 torr vs P_50_(WT) = 2.2 torr)^21^.

Quite surprisingly, the hydrophobic cluster involved in the support of the heme, and including the canonical Phe28(B10), Phe32(B14), Phe42(CD1) and Phe61(E4) is loosened in the Gly-loop mutant. As shown, in Fig. 4D, Phe42 and Phe61 are displaced with respect to the WT Ngb phenylalanine cluster: Phe42 is located about 0.6 Å further away from the heme (C*ε*(Phe42)-C3 methyl(heme) distance), whereas Phe61 is laterally shifted of approximately 2.4 Å (C*ζ*(Phe61 wt)-C*ζ*(Phe61 mutant) distance). These variations are structurally significant, considering that the universally conserved Phe42 is the residue in direct contact with the heme^4,28^, and could constitute an additional factor affecting the heme mobility, possibly contributing to increased binding velocity observed by rapid mixing.

### Evidence of the distal histidine “gating out” movement and of the reshaping of the D-segment upon partial CO binding observed by X-ray crystallography

**Figure 5:**
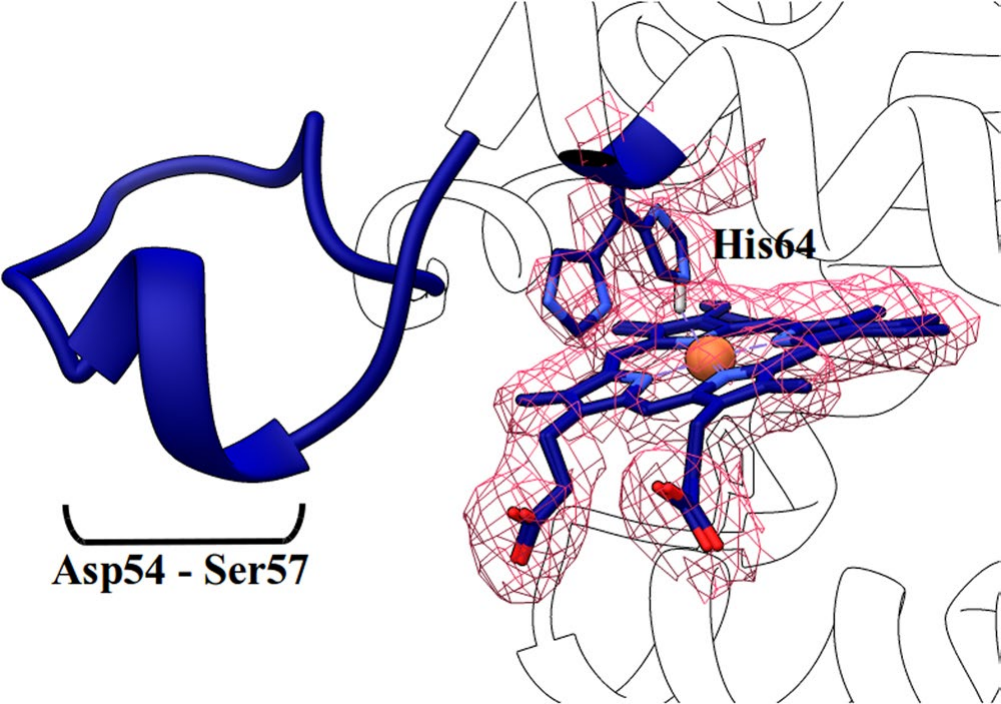
Evidence for His64 swing in Gly-loop structure bound to CO. The electron density indicates the presence of a fraction of swung out His64 (40%, the 2Fo-Fc map is contoured at 1*σ*). We could reconstruct the CD loop and we observed a re-shaping of the D-helix (from Asp54 to Ser57).

Crystals of the Gly-loop mutant tend to dissolve upon exposure to CO in the reduced Fe^2+^ state. However, by freezing crystals before complete disruption, we managed to determine a structure in which we observed about 40% CO ligation and a concomitant swung out histidine conformation (Fig. 5). Moreover electron density appeared for the 49–56 segment that could be successfully built. This indicates that, in the absence of the constraint imposed by the propionate-Tyr44 bond, a state with a swung out His64 can be trapped and structurally characterized, with the heme still in the unslid position. The transition of the CD-loop-D-helix segment is an indication that a remodeling of this region is associated to ligand binding, as also suggested by MD simulations, which is likely the cause for destabilization of the crystal lattice upon full ligation.

### MD Simulations predict the CD loop-D-helix disordering and support the gating out movement of His64 promoting the partial refolding of D-helix

The complete 3D homology model of the Gly-loop mutant modeled with the same fold as WT Ngb (Supplementary Fig. S8) was used as a starting point for MD simulations in order to assess the rearrangement of the CD corner upon the triple glycine substitution and to compare the results with the WT MD. After equilibration using standard MD simulations, we performed simulated tempering MD (ST-MD) of the Gly-loop mutant and of the WT, initially in a range of 300–420 K.

**Figure 6:**
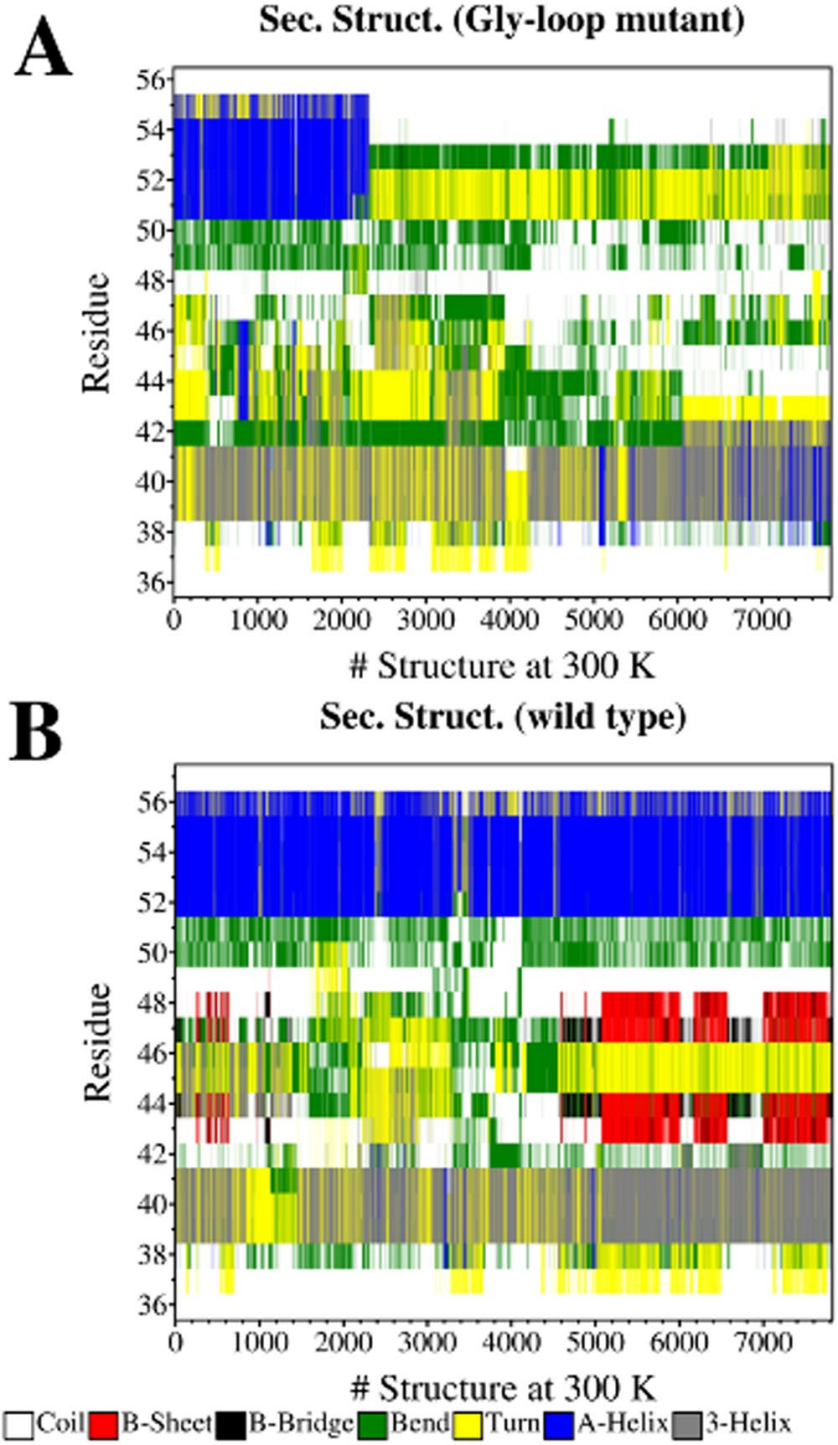
Secondary structure of CD corner at 300 K. The secondary structure was determined by DSSP (hydrogen bond estimation algorithm) on the ensemble of structures, sorted by time in ascending order, at 300 K, for Gly-loop Ngb (**A**) and WT Ngb (**B**).

In Fig. 6, we report the secondary structure of the CD corner at 300 K as a function of time, for the Gly-loop mutant and WT Ngb. The conformation of the C-helix fluctuates during the simulation between a 3-helix and a turn, both in WT and in the mutant, showing that, despite the peptide strand remains in a twisted conformation, the optimal geometry of the hydrogen bond involving the main chain is continuously lost and immediately regained. On the other hand, while the D-helix remains folded during the whole simulation for the WT, in the mutant, the helix unfolds within the first 110 ns and remains unfolded. The reduced structural stability of the D-helix in the Gly-loop mutant is further confirmed by a 60 ns ST-MD simulation in a temperature range of 300–460 K. In that condition, a helix-coil transition in the D-helix was observed after 40 ns for the Gly-loop mutant, then steadily maintained at 300 K, without regaining the helical conformation. Due to high temperatures, a helix-coil transition was observed also for WT Ngb, but the D-helix folded again at the end of the simulation. In Supplementary Fig. S9A, we have reported the most representative configurations (80%), obtained by clustering the structures of the Gly-loop mutant at 300 K. The severe destabilization of the D-helix in the Gly-loop mutant was confirmed by the free energy landscapes that describe the helix unfolding (Supplementary Fig. S10) where the unfolded configurations are more stable of 5–8 kJ/mol than in WT Ngb.

To test the hypothesis that the prevailing effect of the glycine substitutions in the CD corner is to favor the gating out of His64 upon external ligation to the heme, resulting in the observed increased reactivity with CO, we studied the His64 swinging motion, both in the Gly-loop mutant and in WT, choosing the Fe-N distance between the heme and His64 as reaction coordinate. In Supplementary Fig. S11A, we report some snapshots describing the displacement of His64 and the opening of the His-gate. It is worth noting that in Gly-loop Ngb, a relevant structural rearrangement of the CD corner is shown throughout the swing in/out of His64, where the D-helix recovers its folding. Instead, such a rearrangement is not observed in WT, and the CD corner remains folded throughout the His64 displacement.

The free energy landscapes of the His64 displacement (Supplementary Fig. S11B) show that its removal from the sixth coordination position is thermodynamically and kinetically favored in Gly-loop Ngb. This effect is rather moderate up to the distance of 0.4 nm, where the removal of His64 is at least 2 kJ/mol more favorable, but at higher Fe-N distances, the free energy barrier decreases from 16.5 kJ/mol in WT to 6 kJ/mol in the mutant, and the swung-out conformations (corresponding to the open His-gate) are between 5 and 18 kJ/mol more stable in Gly-loop Ngb than in WT Ngb. The difference between the two profiles can be explained considering not only an increased mobility of the CD corner in the mutant but also the existence of a coupling between the gating out movement of the distal His64 and the structural rearrangement of the CD corner, that mainly involves the refolding of the D-helix. Thus, the swung out conformations are promoted by the return of the D-helix to a folded state, and therefore to more enthalpically favored conformations.

## Concluding Remarks

By analyzing the kinetics, spectroscopic and structural features of three Ngb mutants, we have highlighted that the control of external ligand binding, mainly dependent on the spontaneous dissociation of the distal His64 is exerted (i) by the swinging out of distal His64 that depends on Tyr44 and on the dynamics of the CD-loop-D-helix segment and (ii) by the barriers to the heme sliding within its crevice consisting of residues on the proximal side, mostly Phe106^20^.

The effect of the double heme insertion observed in Ngb is enhanced by the increased size of the large Ngb internal cavity created by the F106A mutation, which results in a set of alternate conformations of proximal side residues that account for the enhanced biphasic behaviour in ligand binding of mutants carrying this substitution.

We propose a sequence of events for ligand binding to neuroglobin, in which after spontaneous rupture of the 6th coordination bond of His64 to the heme iron, exogenous diatomic ligands such as CO can be coordinated by the heme only upon swinging out of His64, having isolated and determined the structure of this intermediate in the Gly-loop mutant where the Tyr44-propionate barrier is absent. Heme sliding within the heme crevice follows this intermediate step and leads to partial return of His64 in the distal cavity as indicated by resonance Raman spectroscopy. Removal of the barrier to His64 early swinging out increases the velocity of binding by one order of magnitude, reaching a much faster rate limiting step in the Gly-loop mutant with respect to WT Ngb. Consistently with our hypothesis, this indicates that, in the Gly-loop mutant (where the barrier to His64 gating out is removed and Phe106 still needs to rearrange to allow heme sliding), the fraction of the A_0_ open conformation is predominant. Indeed, in Gly-loop-F106A, where the main barrier to sliding is removed, the A_0_/A_3_ ratio (open/closed) is restored to a value similar to the one observed for WT Ngb.

In the double mutant F106A/Gly-loop we found a more than additive effect of the loosening of both distal and proximal control as shown by the fifty times increase in CO binding rate, which might be due to a global destabilization of the hexacoordinated state (or stabilization of the ligand-bound one) over-and-above the local effect on the 6th coordination position. Raman spectroscopy and MD simulation support our interpretation of the structural and kinetics data.

## Methods

### Cloning, expression and purification

We utilized as WT Ngb the C55S/C120S murine gene^14^. The CD loop “Gly-loop” mutation was introduced by means of the HiFi expand kit in combination with the Gibson Assembly kit (BioLabs). The F106A mutation was introduced using the QuickChange Lightning SDM kit (Stratagene). WT Ngb and mutants were expressed and purified as previously described^29^. Spectroscopy and rapid mixing experiments were performed in 0.1 M HEPES pH 7.4 at 25 °C.

### Resonance Raman spectroscopy

Final protein concentration was in the range of 40–150 *μ*M. Ferrous samples were prepared by adding Na-dithionite (20 mg/mL). The CO derivative was obtained by CO equilibration (^12^CO or ^13^CO (Rivoira, Milan, Italy)), followed by reduction of the heme with Na-dithionite. RR spectra were obtained by excitation with the 413.1 nm line of Kr^+^ laser (Innova 300C, Coherent, Santa Clara, CA, USA) and the 441.6 nm line of a He–Cd laser (Kimmon IK4121R-G). The RR spectra were calibrated with indene, carbon tetrachloride and acetonitrile as standards to an accuracy of 1 cm^−1^ for intense isolated bands. All RR measurements were repeated several times to ensure reproducibility. To improve the signal-to-noise ratio, a number of spectra were accumulated and summed only if no spectral differences were noted. All spectra were baseline-corrected. Absorption spectra were measured both prior to and after RR measurements to ensure that no degradation had occurred (Supplementary Materials and Methods).

### Rapid mixing experiments

Stopped-flow experiments were performed with a 1 cm light path Applied Photophysics apparatus. Ngb solutions and buffers were degassed by N_2_ equilibration. Heme iron reduction from Fe^3+^ to Fe^2+^ (ferric to ferrous) was achieved by anaerobically adding 200 *μ*M Na-dithionite. Solutions of CO concentrated from 1 mM to 15 *μ*M were prepared by consecutive dilutions of the 1 mM CO stock solution with N_2_-equilibrated buffer. The binding reaction between the 10 *μ*M ferrous Ngbs (WT and Gly-loop/F106A) or 5 *μ*M ferrous Ngbs (F106A and Gly-loop) and CO was followed at 426 nm. Rate constants and amplitudes were determined using the Qtiplot software.

### Crystallization, data collection and analysis

Crystals were obtained using the vapor diffusion method in hanging drops. Data collection was carried out at ESRF (France) and Elettra (Italy), and structures were solved by molecular replacement. (Supplementary Table S1).

### Molecular Dynamics (MD) simulations

The initial coordinates for WT Ngb were from PDB entry 1Q1F^14^, for the Gly-loop variant the missing residues Phe49-Ser57 were modeled by homologs. Proteins were solvated with explicit TIP3P waters. All MD simulations were performed with the GROMACS package^30^ version 2016.4 using CHARMM36m force field^31^. The detailed protocol for MD simulations is given in Supplementary Materials and Methods. Simulated tempering (ST)-MD were performed for both systems for 300 ns. The free energies profiles were obtained using umbrella sampling and weighted histogram analysis method (WHAM)^32^. The initial configurations, one for each umbrella window, were generated by steered molecular dynamics (SMD) simulations^33^ coupled with simulated tempering^34^. The configuration for each window was obtained collecting and grouping the configurations at 300 K in sub-ensemble according their projection along the reaction coordinate, and clustering them using the GROMOS method. Starting from the structures reported in Supplementary Fig. S10, an equilibration run of 100 ns was performed. Then the protein was switched to the pentacoordinated state. After an energy minimization, and equilibration of other 100 ns, the Fe-N distance between the heme and the distal histidine was constrained at 2.2 Å during a ST-MD of 10 ns. Then a representative structure at 300 K was chosen and the His64 was pulled away from the iron atom. From that simulation, the configurations for the following umbrella sampling were extracted.

## Supplementary information

Supplementary Info
