# Supplementary material for: Proximal and distal control for ligand binding in neuroglobin: role of the CD loop and evidence for His64 gating
Source: Sci Rep. 2019 Mar 29;9:5326. doi: 10.1038/s41598-019-41780-3 (PMC6441039; doi:10.1038/s41598-019-41780-3)
Supplement: Supplementary file 1 — Supplementary Info [file 41598_2019_41780_MOESM1_ESM.pdf]

Supplementary Information for

Proximal and distal control for ligand binding in neuroglobin: role of the CD loop and evidence for His64 gating

Cécile Exertier[a,b], Lisa Milazzo[c], Ida Freda[a,b], Linda Celeste Montemiglio[a,b,e], Antonella Scaglione[a,b,], Gabriele Cerutti[a], Giacomo Parisi[a,b,], Massimiliano Anselmi[d], Giulietta Smulevich[c], Carmelinda Savino[e,1] and Beatrice Vallone[a,b,e,*]

[a]Dip. di Scienze Biochimiche «A.R. Fanelli», Sapienza Università di Roma, P.le A. Moro 5, 00185 Rome, Italy

[b]Instituto Pasteur-Fondazione Cenci Bolognetti, Dip. di Scienze Biochimiche «A.R. Fanelli», Sapienza Università di Roma, P.le A. Moro 5, 00185 Rome, Italy

[c]Dip. di Chimica «Ugo Schiff», Università di Firenze, Via delle Lastruccia 3-13, 50019 Sesto Fiorentino (FI), Italy

[d]Institute for Microbiology and Genetics, Georg-August University Gottingen, Justus-con-Liebig-Weg 11, 37077 Gottingen, Germany

[e]CNR Institute of Molecular Biology and Pathology, P.le A. Moro 5, 00185, Rome, Italy

*To whom correspondence should be addressed: E-mail: beatrice.vallone@uniroma1.it

The Supplementary Information includes:

Supplementary text
Supplementary Figures S1 to S11
Supplementary Tables S1 to S4
References for Supplementary Information

Supplementary text

Materials and methods

Resonance Raman (RR) - All the samples were in 100 mM Hepes pH 7.4. The ferrous samples were prepared by addition of a small volume (2-5µL) of a freshly prepared sodium dithionite solution (20 mg/mL) to the ferric forms previously flushed with nitrogen. The ferrous-CO complexes were prepared by flushing the ferric forms with ^{12}CO or ^{13}CO (Rivoira, Milan, Italy), and reducing the heme by addition of a small volume (2-5µL) of a freshly prepared sodium dithionite solution (20 mg/mL). All chemicals were of analytical or reagent grade and were used without further purification. Protein concentration in the range 40–150 µM was used for electronic absorption and Resonance Raman (RR) spectroscopies at room temperature. The protein concentration was estimated on the basis of the extinction coefficient of the ferric form at 532 nm, = 10700 M^{-1} cm^{-1} (1).

The RR spectra were obtained at 25 °C using a 5-mm NMR tube by excitation with the 413.1 nm line of a Kr^+ laser (Innova 300 C, Coherent, Santa Clara, CA, USA) and the 441.6 nm line of a He–Cd laser (Kimmon IK4121R-G). Backscattered light from a slowly rotating NMR tube was collected and focused into a triple spectrometer (consisting of two Acton Research SpectraPro 2300i instruments and a SpectraPro 2500i instrument in the final stage with 3600 grooves/mm and 1800 grooves/mm gratings) working in the subtractive mode, equipped with a liquid nitrogen-cooled CCD detector. A spectral resolution of 1.2 cm^{-1} and spectral dispersion of 0.40 cm^{-1}/pixel were calculated theoretically on the basis of the optical properties of the spectrometer for the 3600 grating; for the 1800 grating, used to collect the RR spectra of the ferrous-CO adducts in the 2000–2300 cm^{-1} region, the spectral resolution was 4 cm^{-1} and spectral dispersion 1.2 cm^{-1}/pixel. A cylindrical lens, which focuses the laser beam in the sample to a narrow strip rather than the usual point, was used to collect the spectra of the ferrous-CO adducts in order to avoid photolysis. The RR spectra were calibrated with indene, carbon tetrachloride and acetonitrile as standards to an accuracy of 1 cm^{-1} for intense isolated bands. All RR measurements were repeated several times under the same conditions to ensure reproducibility. To improve the signal-to-noise ratio, a number of spectra were accumulated and summed only if no spectral differences were noted. All spectra were baseline-corrected.

Absorption spectra (Cary 60 spectrophotometer, Agilent Technologies, Glostrup, Denmark, resolution of 1.5 nm) were measured both prior to and after RR measurements to ensure that no degradation occurred under the experimental conditions used.

Crystallization, data collection and data analysis - Prior to data collection, unliganded Ngb crystals were cryo-protected with 20% glycerol and flash frozen in liquid nitrogen. To obtain the structure of the CO-bound forms, crystals were first soaked in CO-saturated mother liquor for 30 minutes and then in CO-saturated mother liquor containing also 50mM sodium dithionite and 20% glycerol to proceed with iron reduction, and CO binding.

The same procedure was adopted for F106A and Gly-loop CO-bound structures. No significant structural rearrangement is induced by the heme iron reduction (2). Crystals of the ferric Gly-loop mutant were soaked for 24 hours in the same crystallization condition, except that ammonium sulfate was substituted with lithium sulfate. Subsequently crystals were exposed to 8% glutaraldehyde vapors for two hours, prior to reduction and CO soaking as previously described. Data collection information and final refinement statistics are reported in SI Appendix **Table S1**. Structures were solved by molecular replacement using the pdb entry 1Q1F for the ferric form of proteins, and the pdb entry 1W92 for the liganded form using MolRep (3). Refinement and model building were carried out using the ccp4i package (4) including Refmac5 (5) and Coot (6) respectively. Images were made using UCSF Chimera (7).

MD simulations - The initial coordinates for wild type were taken from the 1.5 Å x-ray structure of murine ferric bis-histidine Ngb (PDB entry 1Q1F) (8). Lacking residues at the amino terminus were

added using other crystallographic structures as template, whereas the C-ter residue Glu151 was added using Pymol software package (9). For a better comparison with the experimental data, the mutations C55S and C120S, introduced for favoring crystallization, were maintained. The heme conformer A with the highest occupancy was chosen, and the iron atom was considered in ferrous state. The initial coordinates for the Gly-loop mutant were obtained from its crystal structure, after removing the residue His0 at the amino terminus and adding the residue Glu150. The region between the helices B and E, comprising the helices C and D and the connecting loops, was initially determined by the SWISS-MODEL protein structure homology-modelling server (10), using as template the coordinates of the peptide strand 33-62 in wild type. Each protein molecule was put at the center of a dodecahedron box, large enough to contain the protein and at least 0.9 nm of solvent on all sides. The protein was solvated with explicit TIP3P water molecules (11). The water molecules eventually inserted in the hydrophobic cavities inside the protein matrix were removed. All MD simulations were performed with the GROMACS software package (12) version 2016.4 using CHARMM36m force field (13). Long range electrostatic interactions were calculated with the particle-mesh Ewald scheme (14). A cutoff of 1.2 nm was applied to the direct-space Coulomb and Lennard-Jones interactions. The bond lengths and angles of water molecules were constrained with the SETTLE algorithm (15), and all other bonds were constrained with LINCS (16). The pressure was set to 1 bar using the Parrinello-Rahman barostat (τ = 5 ps) (17). The temperature was fixed at 300 K using velocity rescaling with a stochastic term (τ = 2 ps) (18). For all systems, the solvent was relaxed by energy minimization followed by 100 ps of MD at 300 K, while restraining protein atomic positions with a harmonic potential. The systems were then minimized without restraints and their temperature brought to 300 K in 10 ns in a stepwise manner. Then the systems were equilibrated at 300 K for 100 ns. In order to ensure an enhanced sampling, simulated tempering (19) MD (ST-MD) simulations were performed for both systems. During the ST-MD, the temperature of the system is changed periodically. At low temperatures, the system is easily trapped in local minima, and increasing the temperature helps the system escape. Temperature changes was controlled according to the Metropolis algorithm (20), so that to obtain in the end canonical ensembles at all the chosen temperatures. Because in ST-MD one needs to assign weights to the temperatures in order to ensure uniform sampling of the temperature space, the initial guess weights were chosen according the procedure described by Pande and al. (21). Starting with these weights, we performed ST-MD for 300 ns. Temperature transitions were attempted every 1 ps, and the weights were updated throughout the whole simulation according to the Wang-Landau adaptive weighting scheme (22). The temperatures were linearly interpolated, considering temperature values every 10 K. The free energies profiles were obtained using umbrella sampling and weighted histogram analysis method (WHAM) (23). The initial configurations, one for each umbrella window, were generated by a steered molecular dynamics (SMD) simulation (24) coupled with simulated tempering (19). In SMD simulations the system is pulled by means of a harmonic potential along a given reaction coordinate. The pull force was set to 1000 kJ/mol nm^2 whereas the reference position was changed with a rate of 5e-6 nm/ps. The coupling with simulated tempering guaranteed a suffice sampling to ensure that the system may be equilibrated throughout the pulling and it may be possible finding a reasonable low energy reaction pathway. The configuration for each window was obtained by means of a cluster analysis, performed on the configurations belonging to the ensemble at 300 K. The configurations, collected and grouped in sub-ensemble according their projection along the reaction coordinate, were clustered using the GROMOS method (25) and the cutoff was chosen on the basis of the root mean square deviation distribution in each sub-ensemble, picking the value corresponding to the first in abscissa relative maximum. All calculations on bis-histidine Ngb were performed using the original parameters of the force field. Then the topology was switched to a pentacoordinated deoxy form, the covalent bond between heme and the distal histidine was removed. The partial charges on the heme and on the proximal histidine were changed, as well as the Fe-N distance between the heme and the proximal histidine, which was set to 2.11 Å, in agreement with XANES (26) experimental data. Finally, a Lennard-Jones potential was added to the iron ion (σ = 0.241 nm and ε = 0.03941 kJ/mol) (27). To determine

the partial charges of the pentacoordinated deoxy Ngb, we performed quantum chemical calculations on the isolated 4-methylimidazole iron^{II} protoporphyrin IX without carboxylic functional groups. Density functional calculations with B3LYP (28, 29) were performed. We used the LANL2TZ basis set (30) for iron and 6-311++G(d,p) basis set (31) for all other atoms. After a geometry optimization and a single point calculation, the partial charges were obtained by means of the restrained electrostatic potential (RESP) procedure (32), fitting the electrostatic potential calculated around the molecules according the CHELPG scheme (33). All QM calculations were performed using the Gaussian 09 software package (34). Starting from the structures reported in **Fig. S9**, an equilibration run of 100 ns was performed. Then the protein was switched to the pentacoordinated state. After an energy minimization, the system was equilibrated for other 100 ns. The Fe-N distance between the heme and the distal histidine was then constrained at 2.2 Å during a ST-MD of 10 ns. Then a representative structure at 300 K was chosen and a SMD simulation, in which the distal histidine was pulled away from the iron atom, was performed. From that simulation, the configurations for the following umbrella sampling were extracted.

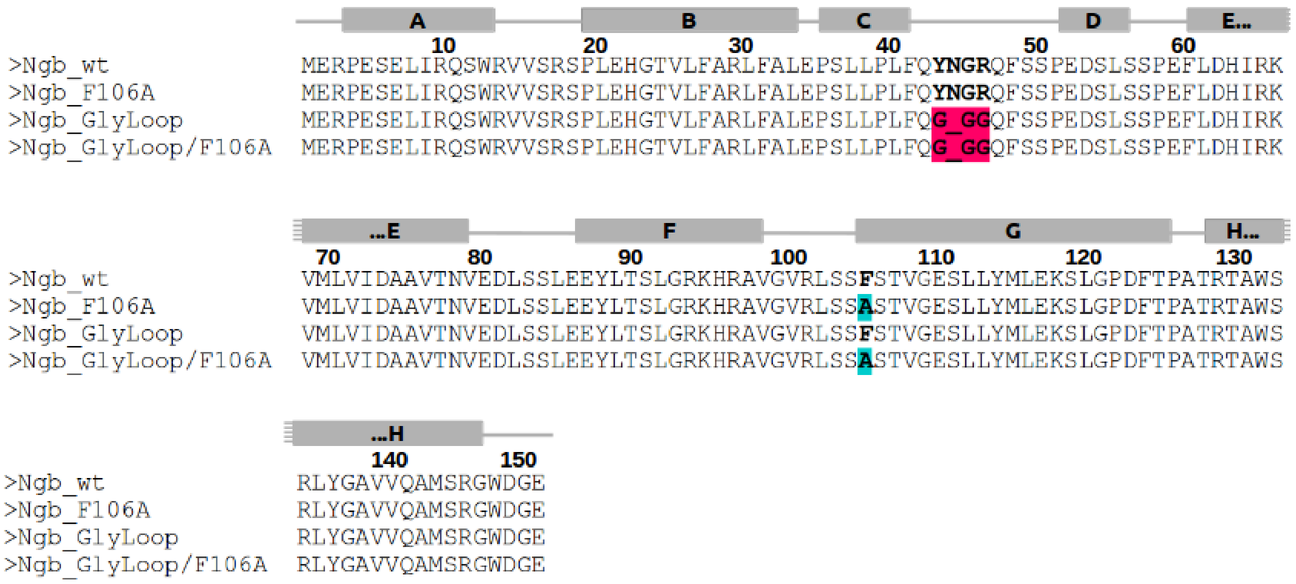

Figure S1. Sequence alignment of neuroglobin mutants. F106A is the mutant containing an alanine (blue) in position 106, where originally was present a phenylalanine in the wild type protein. The Gly-loop mutant is a CD corner mutant, in which part of the CD corner (from Tyr44 to Arg47 in the Ngb wt) was mutated in Gly-Gly-Gly as indicated in pink. The Gly-loop /F106A mutant contains both mutations.

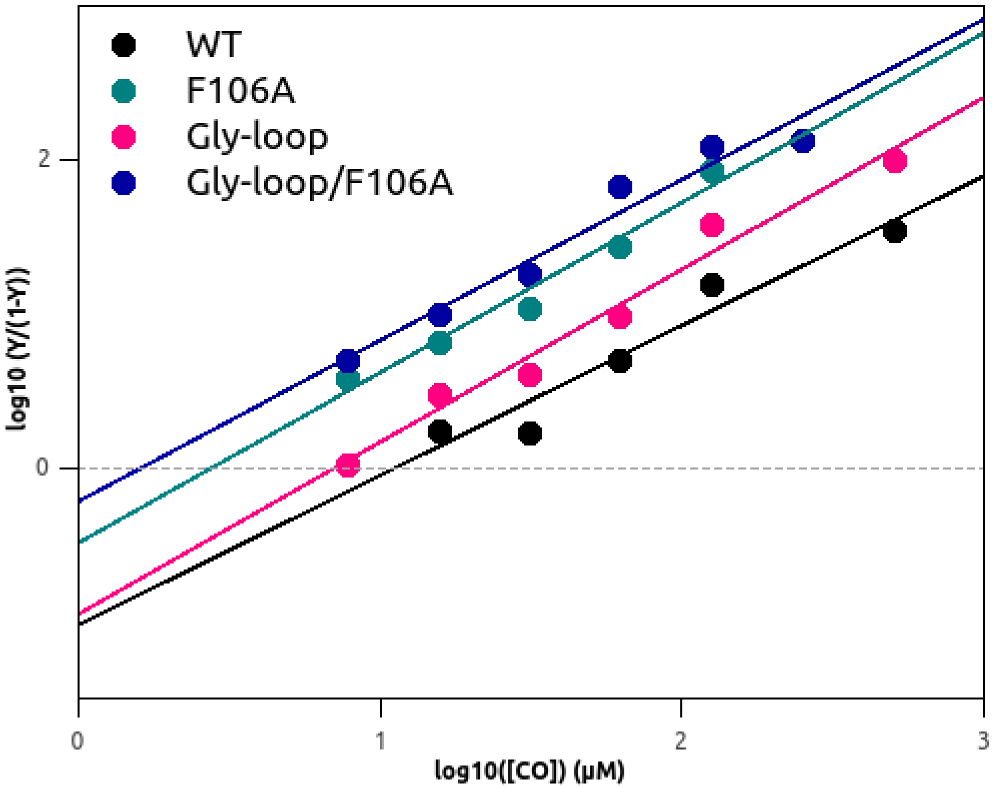

Figure S2. Determination of overall CO binding c_{50} for neuroglobin wild type and mutants at 25°C. The fraction Y corresponding to the fraction of Ngb bound to CO was extracted from the overall amplitudes of the kinetic traces at 500 µM (Rapid mixing experiment, **Fig. 1**), and $\log_{10}(Y/1-Y)$ was plotted as a function of the concentration of CO. Data were linearly fitted to determine the c_{50}.

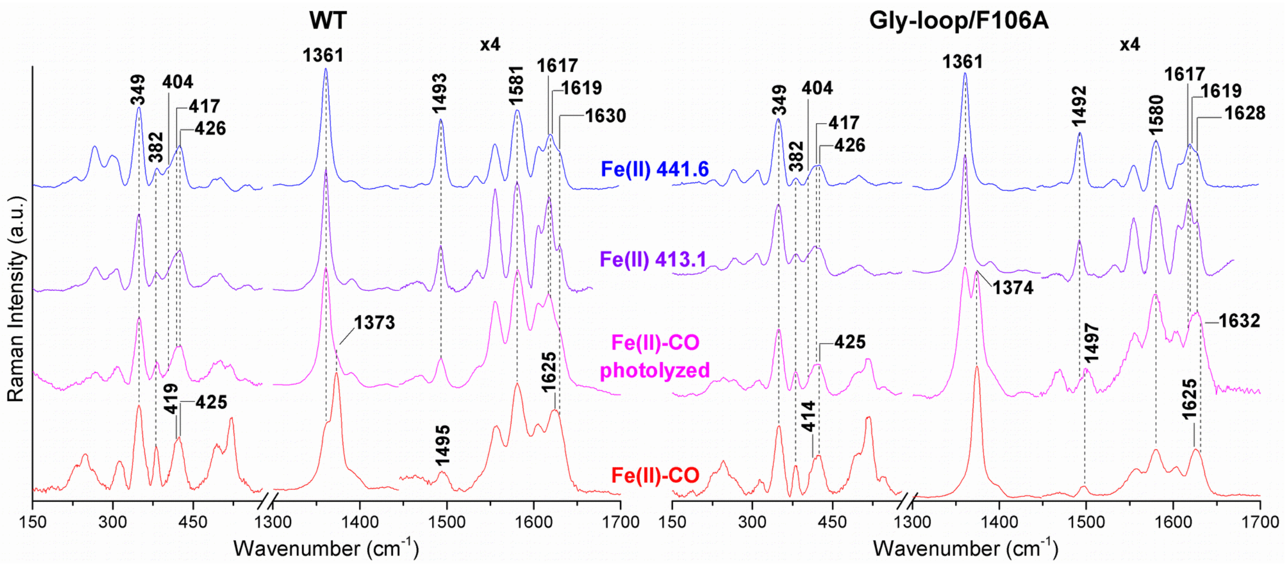

Figure S3. RR spectra of WT (left) and B/F106A mutant (right) NGbs. Low and high frequency regions obtained with the 413.1 nm excitation wavelength of the Fe(II)-CO adduct (red), photolyzed Fe(II)-CO adduct (magenta) and Fe(II) form, (violet). The RR spectrum of the Fe(II) (blue) form obtained with the 441.6 nm excitation wavelength is also reported for comparison, to ensure that no 5cHS is present. The spectra have been shifted along the ordinate axis to allow better visualization. Experimental conditions: Fe(II)-CO: laser power at the sample 1 mW, average of 2 spectra with 20 min integration time (WT and Gly-loop/F106A); Fe(II)-CO photolyzed: laser power at the sample 10 mW, average of 6 spectra with 6 min integration time (WT) and laser power at the sample 20 mW, 1 spectrum with 1 min integration time (Gly-loop/F106A); Fe(II): 413.1 nm excitation wavelength, laser power at the sample 10 mW; average of 6 spectra with 6 min integration time (grating with 1800 grooves per mm) and average of 4 spectra with 20 min integration time (grating with 3600 grooves per mm) in the low and high frequency regions, respectively (WT); average of 6 spectra with 6 min integration time (grating with 1800 grooves per mm) and average of 2 spectra with 20 min integration time (grating with 3600 grooves per mm) in the low and high frequency regions, respectively (Gly-loop/F106A); 441.6 nm excitation wavelength, laser power at the sample 20 mW; average of 5 spectra with 15 min integration time (grating with 1800 grooves per mm) and average of 5 spectra with 10 min integration time (grating with 1800 grooves per mm) in the low and high frequency regions, respectively (WT and B/F106A).

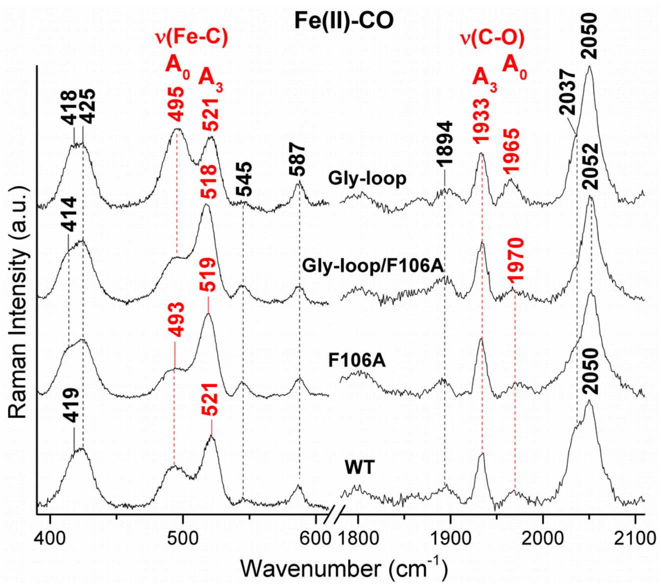

Figure S4. RR spectra in the low (left) and high (right) frequency regions of the ^{12}CO adducts of the WT, F106A, Gly-loop/F106A and Gly-loop mutant Ngbs. The frequencies of the ν(FeC) and ν(CO) modes are indicated in red. The spectra have been shifted along the ordinate axis to allow better visualization. Experimental conditions: 413.1 nm excitation wavelength; laser power at the sample 1 mW, average of 19 spectra with 190 min integration time and 14 spectra with 140 min integration time in the low and high frequency regions, respectively (WT); laser power at the sample 2 mW, average of 9 spectra with 90 min integration time and 16 spectra with 160 min integration time in the low and high frequency regions, respectively (F106A) and average of 7 spectra with 70 min integration time and 16 spectra with 160 min integration time in the low and high frequency regions, respectively (Gly-loop); laser power at the sample 5 mW, average of 8 spectra with 80 min integration time and 9 spectra with 90 min integration time in the low and high frequency regions, respectively (Gly-loop/F106A).

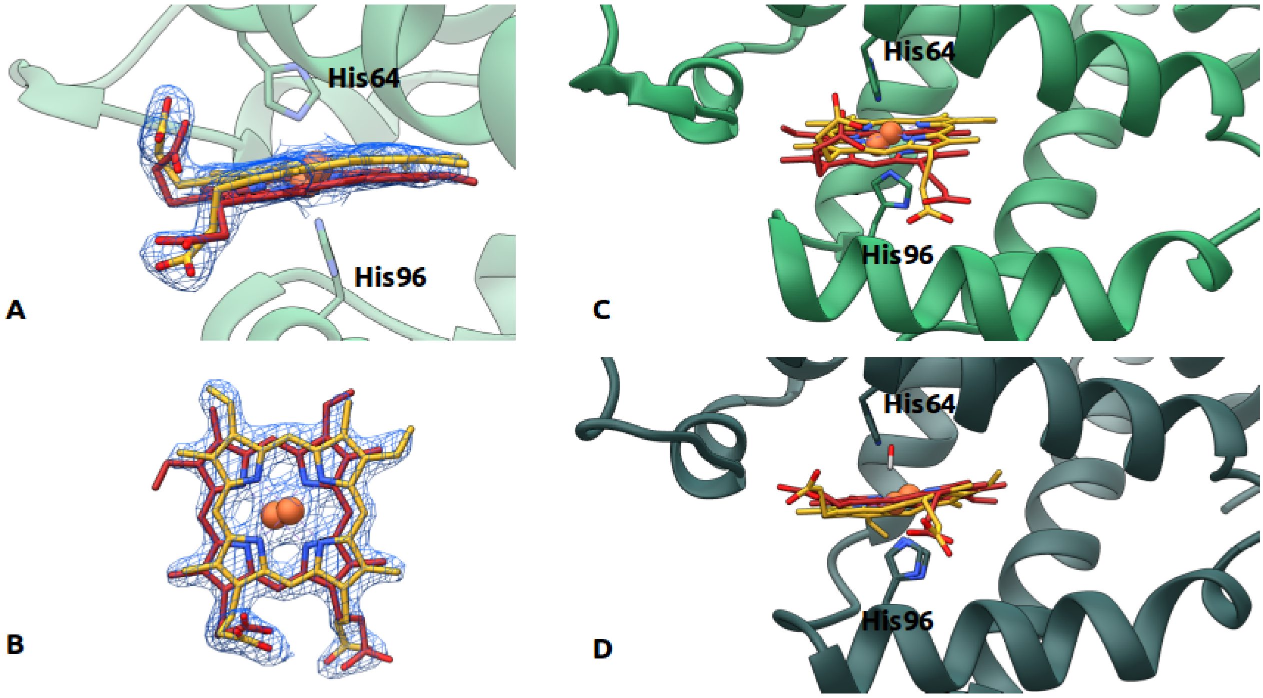

Figure S5. Structures and double heme conformation in F106A neuroglobin observed by X-ray crystallography.
A and **B,** view of the double heme conformation in hexacoordinated F106A (The 2Fo-Fc map is contoured at 1σ). In red, the heme conformer B present at 25% (canonical heme orientation in myoglobins), in yellow the major heme conformer A (reversed heme). **C** and **D** shows the relative positions of the heme double conformations respectively in ferric F106A and F106A-CO structures.

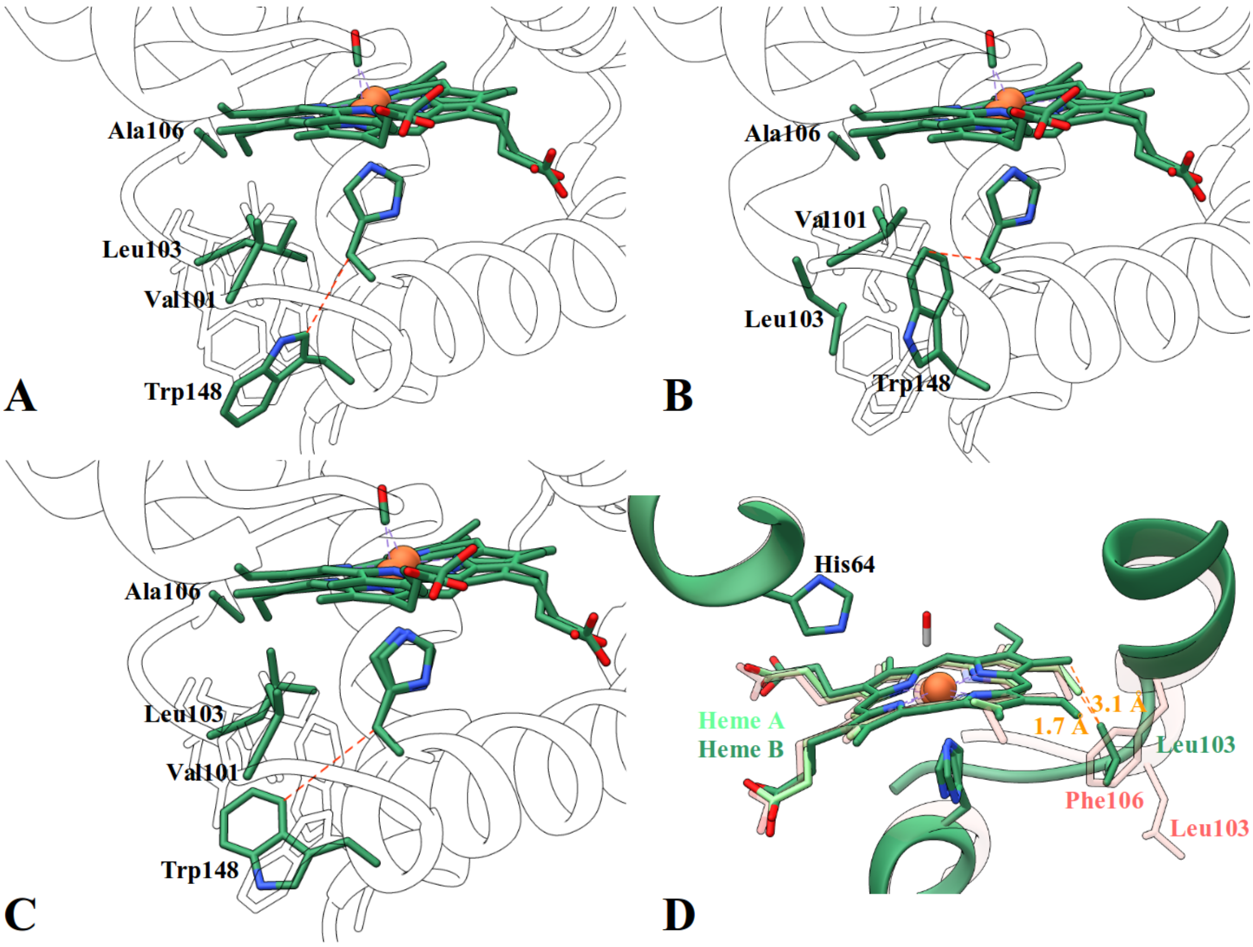

Figure S6. Consequence of the presence of an alanine in position 106 on the conformation of the proximal heme environment in murine neuroglobin bound to CO. **A,B,C,** The FG segment adopts multiple conformations upon CO binding. **D**. Upon CO binding, Leu103 is repositioned in the niche occupied by Phe106 in WT, as sterically allowed only by the heme conformer B. We may ascribe the increased mobility of Trp148 to the loss of the offset π-stacking present in WT Ngb between Phe106 and Trp148, that allows Trp148 displacement as the C_β of proximal His96 leans on it upon heme sliding. One of the alternate conformations assumed by Trp148, upon His64 pressure, induces the flipping of Leu103 towards the bulk, whereas another conformer forces Leu103 in the niche occupied in the wild type by Phe106. The latter conformation is sterically allowed only by the B heme conformer, due to the reduced size of the methyl moiety (**Fig. S6D**). Ala106, Trp148 and the FG loop (from Val99 to Ser105) are spatially close in the proximal cavity of the heme, and the above mentioned set of events triggers the repositioning of the FG loop (**Fig. S6C**).

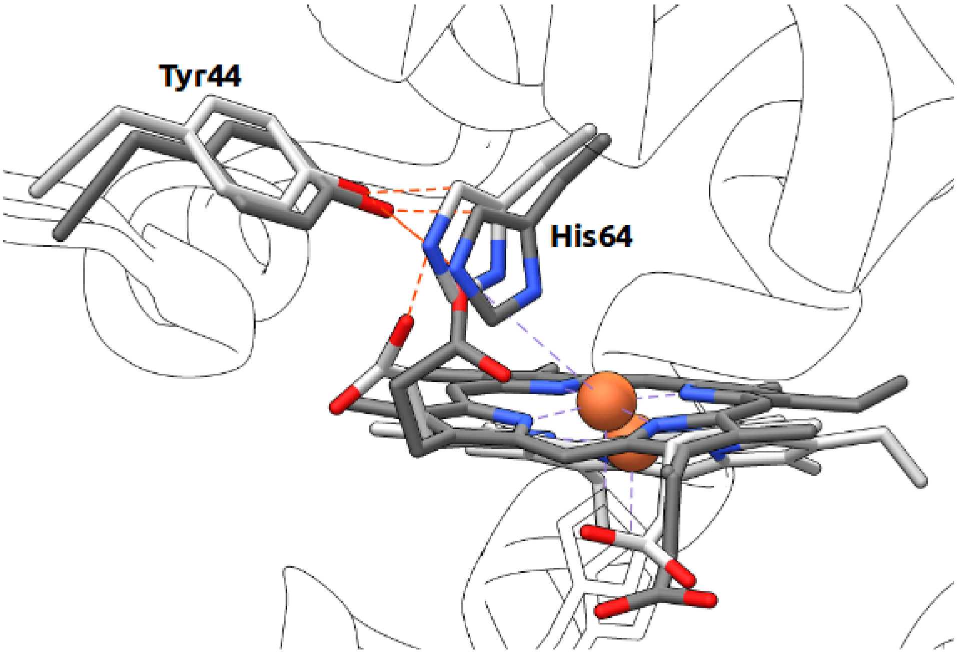

Figure S7. Simulation of His64 swinging out movement in the crystal structure of neuroglobin wild type. In ferric Ngb (dark grey,1Q1F (8)) and Ngb-CO (light grey, 1W92, (35)), the distal histidine seems to clash against Tyr44 and the heme propionate in the crystal.

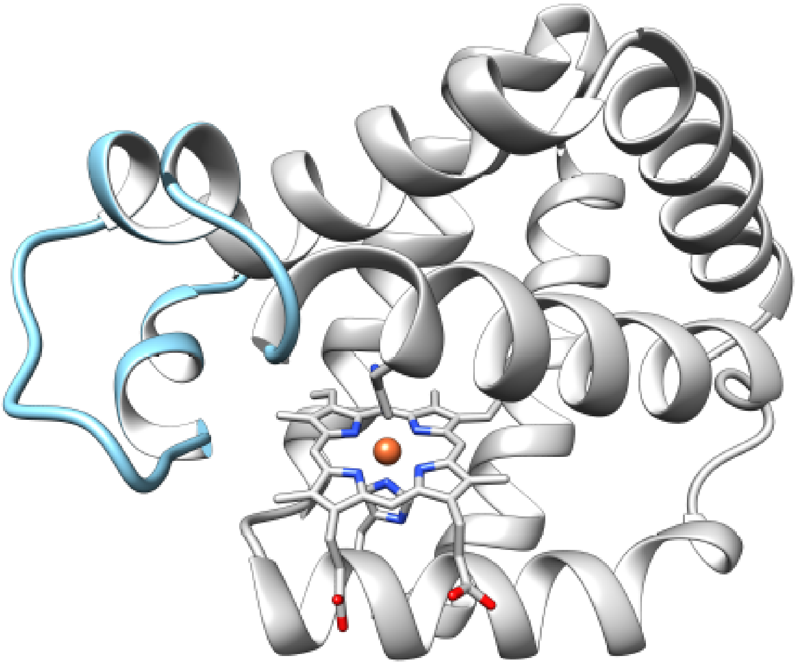

Figure S8. Three-dimensional homology model of the Gly-loop mutant. The coordinates were achieved using the WT structure as a template, and they served as a starting point for MD simulations. The previous procedure assigned to the CD corner of the Gly-loop mutant, the same folding as observed in WT Ngb. Starting from a complete folded structure instead of a partially unfolded one, allowed the assessment of the structural rearrangement in the CD corner upon triple glycine substitutions and the direct comparison of the results with the WT protein.

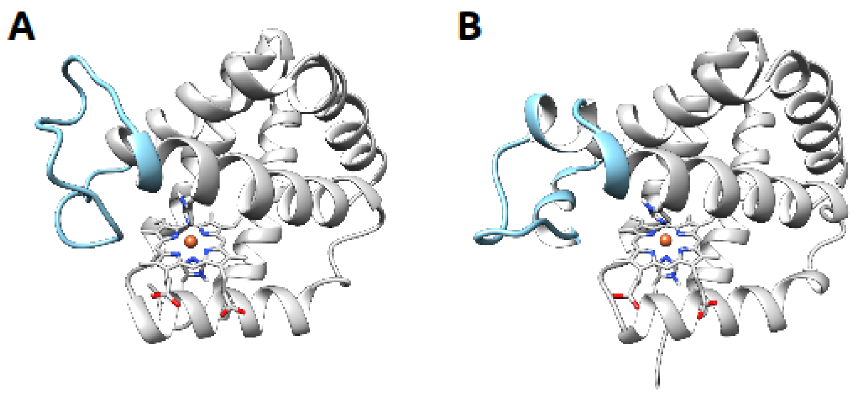

Figure S9. Representative configurations of the Gly-loop mutant and the WT Ngb as obtained by simulated tempering. The first representative configurations were obtained by clustering the structures of the Gly-loop mutant (**A**) and of the WT Ngb (**B**) at 300 K. The representative structure of the mutant was picked from the central structure of the main cluster including ~80% of configurations. Analogously, the first most populated cluser of the WT encloses ~90% of the configurations.

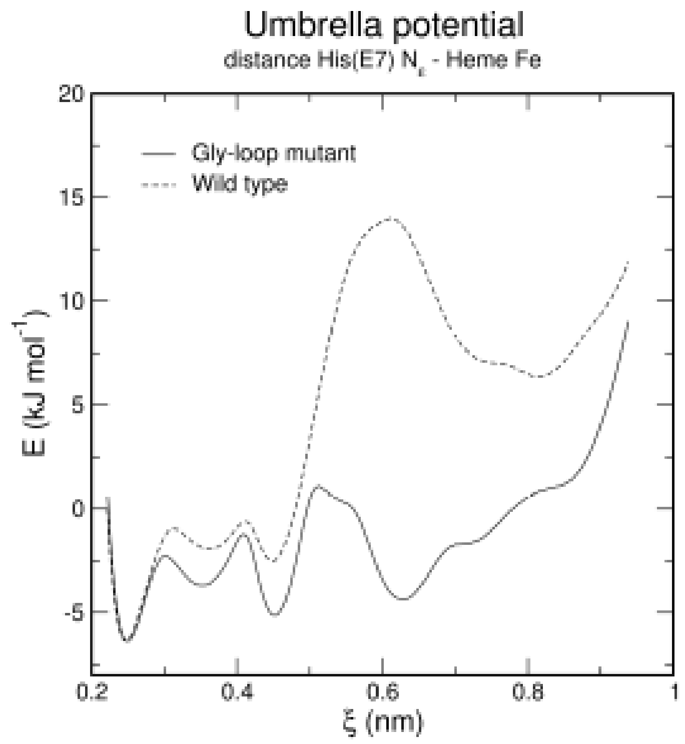

Figure S10. Umbrella Sampling: unfolding of the D-helix

The reaction coordinate is the distance between the C atoms of residues Glu(D2) and Ser(D6), both belonging to the helix D. Such a distance was chosen because in previous calculations it was shown to describe very well the folding-unfolding transition with two distinct, well separated, basins. For the calculation, 35 equi-spaced windows, spanning the distance range from 0.62 to 1.3 nm, were used, and a single simulation run (10 ns) was performed for each window. In both profiles the global-minimum free energy corresponds to the native conformation of the helix in folded state, exactly as in the "folding funnel" model (36). Being the profile a mono-dimensional representation of a multidimensional landscape, the conformational entropy dramatically increases, going from lower distances to higher distances. Because the number of accessible configurations in extended conformation is much higher, their contribution in folding-unfolding equilibrium becomes crucial, upon their stabilization, regarding the Gly-loop mutant.

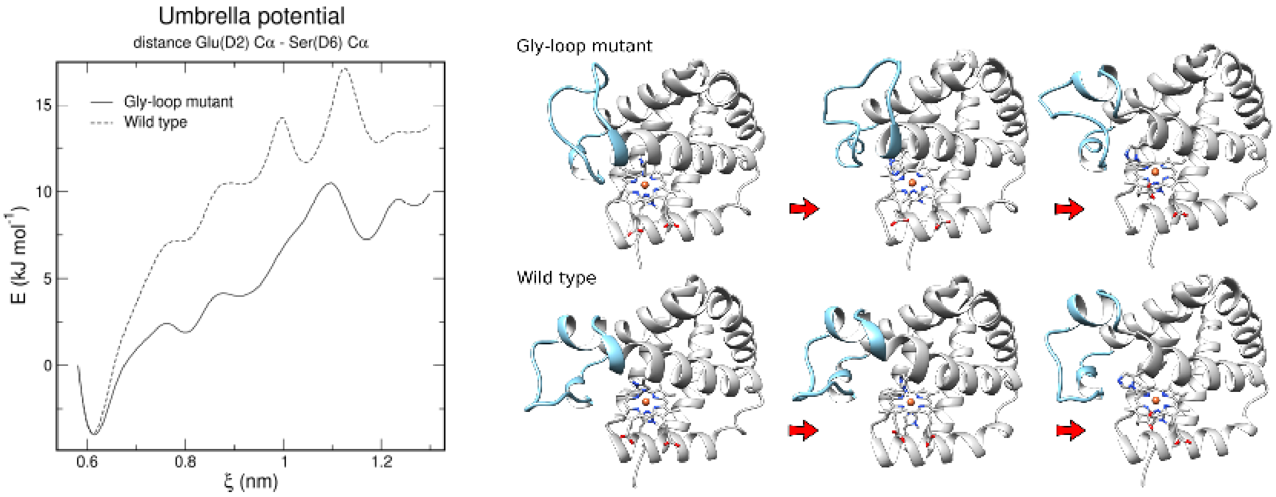

A **B**

Figure S11. Umbrella Sampling: distal histidine displacement. In **A**, snapshots describing the displacement of the distal histidine and the opening of the His-gate. In Gly-loop mutant, a relevant structural rearrangement of the CD corner is shown throughout the swing of the distal histidine, with the helix D that recovers its native folding. Instead, such a rearrangement is not observed in WT, and the CD corner remains folded throughout the distal histidine displacement. In **B**, the energetic landscapes of the distal His64 displacement calculated by umbrella sampling, using 37 equi-spaced windows spanning the distance range from 0.22 to 0.94 nm, and performing 6 ns simulation run for each window. Note that the classical potential used cannot consider the binding reaction of the distal histidine to the heme at shortest distances.

	F106A	F106A-CO	Gly-loop	Gly-loop-CO
pdb code	6H5Z	6H6C	6H6I	6H6J
Crystallization conditions	1.6 M ammonium sulfate, 0.1 M MES pH 6.5, 10% 1,4-dioxane	1.6 M ammonium sulfate, 0.1 M MES pH 6.5, 10% 1,4-dioxane	1.6 M ammonium sulfate, 0.1 M Tris pH 7.5, 4% PEG1000	1.6 M ammonium sulfate, 0.1 M Tris pH 7.5, 4% PEG1000
**Data collection**				
Beamline	Elettra XRD1	ESRF ID29	ESRF ID30B	Elettra XRD1
Wavelength(A)	1	1.07	0.97	1
(°)	0.5	0.2	0.1	1
Number of images	720	1800	1300	220?
Space group	R32	R32	$P3_1 2 1$	$P3_1 2 1$
Unit cell parameters (Å/°)	87.7/87.7/113.6	88.9/88.9/110.3	74.8/74.8/77.1	74.9/74.9/77.2
	90/90/120	90/90/120	90/90/120	90/90/90
Resolution range (A)	45.54 – 1.80 (1.91 - 1.80)	63.15 – 1.75 (1.96-1.75)	33.65 – 1.57 (1.68-1.57)	38.60 – 2.60(2.75 – 2.60)
No of reflexions	313893 (50346)	579229 (92020)	260411 (49232)	90675 (14207)
No of unique reflections	30243(4888)	29354 (4692)	35244 (6429)	15006 (2390)
Completeness (%)	99.9 (99.7)	100 (99.8)	99.8 (100)	99.4 (97.8)
I/sigma (I)	33.62 (2.83)	13.2 (0.47)	18.23 (1.63)	3.10 (0.51)
CC1/2	100.0 (87.2)	99.9 (32.2)	99.9 (65.6)	90.3 (10.4)
Mosaicity	0.243	0.101	0.115	0.288
**Refinement**				
Wilson B-factors (Å^2)	35.2	26.3	23.5	44.1
Rwork/Rfree	0.152/0.209	0.131/0.210	0.137/0.180	0.232/0.266
Used Reflections	15044	16336	35206	7661
Number of atoms	1596	1756	1663	1419
Protein	1431	1513	1394	1220
Heme/CO	85/0	85/2	85/0	85/2
PEG	-	-	27	21
Sulfate ions	5	10	5	10
Tris	-	-	8	8
Glycerol	-	12	12	12
Dioxane	6	18	-	-
Water molecules	67	119	129	54
**Mean B factors (A^2)**				
Protein	40.93	30.7	33.68	42.71
Heme (and CO)	22.68	19.7	28.53	33.89
Solvent (Waters, others)	47.34/59.02	41.70/38.50	50.60/65.30	43.00/67.30
rmsd bond length (A)	0.0174	0.0162	0.0189	0.0120
rmsd angles (°)	1.970	1.879	2.089	1.873
**Ramachandran plot statistics**				
Favored (%)	97.9	98.7	99.3	98.6
Allowed (%)	1.4	1.3	0.7	1.4
Outliers (%)	0.7 (K119)	0	0	0

Table S1. Cystallization, Data Collection, Refinements, Statistics and Validation. Statistics regarding the last resolution shell are reported into parenthesis.

	k (s^{-1}) at 500 µM (Amplitude fraction)	c_{50} (µM)
**WT**	0.19 ± 8.1e-4 (1)	13.2 (R^2 = 0.94)
**F106A**	k_{slow} =0.51 ± 7.1e-3 (0.36)<br>k_{fast} = 1.7 ± 2.0e-2 (0.64)	2.8 (R^2 = 0.97)
**Gly-loop**	2.8 ± 8.1e-3 (1)	7.1 (R^2 = 0.98)
**Gly-loop/F106A**	k_{slow} =2.2 ± 3.8e-2 (0.13)<br>k_{fast} = 9.5 ± 5.0e-2 (0.87)	1.6 (R^2 = 0.96)

Table S2. CO binding rate constants at 500 µM and c_{50} determined at 25°C by rapid mixing for neuroglobin wild type and mutants. The correlation factor R^2 accounts for the quality of the fit to experimental data from Figure S1. We report the amplitudes as fractions, since as reported in the Materials and Methods section, concentrations before mixing were : 10 µM for WT Ngb and Gly-loop/BF106A, and 5µM for F106A and Gly-loop mutants. In all cases we recovered 100% of the expected optical transition for the ferrous hexacoordinated to CO bound Ngb.

	**(A_0)**<br>**Open form**<br>**No H-bond**		**(A_3)**<br>**Closed form**<br>**Strong H-bond**	
**Ngb**	ν_{Fe-C}	ν_{CO}	ν_{Fe-C}	ν_{CO}
C. aceratus [36]	489	1965	522	1934
D. mawsoni [36]	489	1965	522	1934
Mouse [37]	492	1969	523	1933
Human [38,39]	494	1972	521	1932
Murine WT[this work]	493	1970	521	1933
Murine F106A[this work]	493	1970	519	1933
Murine Gly-loop[this work]	495	1965	518	1933
Murine Gly-loop/F106A[this work]	495	1970	518	1933
**Mb** (Sperm Whale) [41]	493	1965	517	1932

Table S3. ν(Fe-C) and ν(C-O) stretching frequencies of various Ngbs: *C. aceratus* and *D. mawsoni* Antarctic fishes (37) (light blue), mouse (38) (light brown), human (39, 40) (violet), murine WT (red), F106A (magenta), Gly-loop (grey) and Gly-loop/F106A (green) mutants. The corresponding data of sperm whale Mb are also reported (41, 42) (black). The human Ngb and sperm whale Mb show also a third weak H-bonded conformer (A_1) at 505/1956 and 508/1946 cm^{-1}, respectively, not reported in the table.

	**WT1**		**WT-CO2**	**F106A**		**F106A-CO**		**Gly-loop**	
**Heme conformer**	A	B	-	A	B	A	B	A	B
**Fe-His64 (Å)**	1.9	2.1	-	2.1	2.5	-	-	2.2	1.9
**Fe-His96 (Å)**	2.2	1.9	2.1	2.3	1.9	2.6	2.6	2.0	2.1
**Angle (°)**	177	156	-	159	142	-	-	173	160

Table S4. Distances and angles between the heme and the proximal/distal histidines for each conformers of WT (8, 35), F106A and Gly-loop.

References

1. Fago A, et al. (2004) Allosteric regulation and temperature dependence of oxygen binding in human neuroglobin and cytoglobin: Molecular mechanisms and physiological significance. *J Biol Chem* 279(43):44417–44426.

2. Arcovito A, et al. (2008) An X-ray diffraction and X-ray absorption spectroscopy joint study of neuroglobin. *Arch Biochem Biophys* 475(1):7–13.

3. Vagin A, Teplyakov A (2010) Molecular replacement with MOLREP. *Acta Crystallogr Sect D Biol Crystallogr* 66(1):22–25.

4. Potterton E, Briggs P, Turkenburg M, Dodson E (2003) A graphical user interface to the CCP4 program suite. *Acta Crystallogr - Sect D Biol Crystallogr* 59(7):1131–1137.

5. Murshudov GN, et al. (2011) REFMAC5 for the refinement of macromolecular crystal structures. *Acta Crystallogr Sect D Biol Crystallogr* 67(4):355–367.

6. Emsley P, Lohkamp B, Scott WG, Cowtan K (2010) Features and development of Coot. *Acta Crystallogr Sect D Biol Crystallogr* 66(4):486–501.

7. Pettersen EF, et al. (2004) UCSF Chimera—A Visualization System for Exploratory Research and Analysis. *J Comput Chem* 25:1605–1612.

8. Vallone B, Nienhaus K, Brunori M, Nienhaus GU (2004) The structure of murine neuroglobin: Novel pathways for ligand migration and binding. *Proteins Struct Funct Genet* 56(1):85–92.

9. Schrödinger LLC (2016) The PyMOL Molecular Graphics System. *Schrödinger LLC* Version 1.:http://www.pymol.org.

10. Waterhouse A, et al. (2018) SWISS-MODEL: homology modelling of protein structures and complexes. *Nucleic Acids Res*:gky427.

11. Jorgensen WL, et al. (1983) Comparison of simple potential functions for simulating liquid water Comparison of simple potential functions for simulating liquid water. *J Chem Phys,* 79:926.

12. Van Der Spoel D, et al. (2005) GROMACS: Fast, flexible, and free. *J Comput Chem* 26(16):1701–1718.

13. Huang J, et al. (2016) CHARMM36m: An improved force field for folded and intrinsically disordered proteins. *Nat Methods* 14(1):71–73.

14. Darden T, York D, Pedersen L (1993) Particle mesh Ewald: An N·log(N) method for Ewald sums in large systems. *J Chem Phys* 98(12):10089–10092.

15. Miyamoto S, Kollman PA (1992) Settle: An analytical version of the SHAKE and RATTLE algorithm for rigid water models. *J Comput Chem* 13(8):952–962.

16. Hess B, Bekker H, Berendsen HJC, Fraaije JGEM (1997) LINCS: A Linear Constraint Solver for molecular simulations. *J Comput Chem* 18(12):1463–1472.

17. Parrinello M, Rahman A (1981) Polymorphic transitions in single crystals : A new molecular dynamics method Polymorphic transitions in single crystals : A new molecular dynamics method. *J Appl Phys* 52(12):7182–7190.

18. Bussi G, Donadio D, Parrinello M (2007) Canonical sampling through velocity rescaling. *J Chem Phys* 126(1). doi:10.1063/1.2408420.

19. Marinari E, Parisi G (1992) Simulated tempering: A New Monte Carlo Scheme. *EPL* 19(6):451–458.

20. Metropolis N, Rosenbluth A, Rosenbluth M, Teller A, Teller E (1953) Equation state calculations by fast computing machines. *J Chem Phys* 21(6):1087–1092.

21. Park S, Pande VS (2007) Choosing weights for simulated tempering. *Phys Rev E - Stat Nonlinear, Soft Matter Phys* 76(1). doi:10.1103/PhysRevE.76.016703.

22. Wang F, Landau DP (2001) Efficient, multiple-range random walk algorithm to calculate the density of states. *Phys Rev Lett* 86(10):2050–2053.

23. Kumar S, Rosenberg JM, Bouzida D, Swendsen RH, Kollman PA (1992) THE weighted histogram analysis method for free-energy calculations on biomolecules. I. The method, THE weighted histogram analysis method for free-energy calculations on biomolecules. I. The method. *J Comput Chem J Comput Chem* 13, 13(8, 8):1011, 1011–1021, 1021.

24. Izrailev S, et al. (1999) Steered Molecular Dynamics. *Computational Molecular Dynamics: Challenges, Methods, Ideas*, pp 39–65.

25. Daura X, et al. (1999) Peptide Folding: When Simulation Meets Experiment. *Angew Chemie Int Ed* 38(1/2):236–240.

26. D'Angelo P, et al. (2010) Dynamic investigation of protein metal active sites: Interplay of XANES and molecular dynamics simulations. *J Am Chem Soc* 132(42):14901–14909.

27. Li P, Roberts BP, Chakravorty DK, Merz KM (2013) Rational design of particle mesh ewald compatible lennard-jones parameters for +2 metal cations in explicit solvent. *J Chem Theory Comput* 9(6):2733–2748.

28. Becke AD (1993) Density-functional thermochemistry. III. The role of exact exchange. *J Chem Phys* 98(7):5648–5652.

29. Lee C, Yang W, Parr RG (1988) Development of the Colle-Salvetti correlation-energy formula into a functional of the electron density. *Phys Rev B* 37(2):785–789.

30. Hay PJ, Wadt WR (1985) *Ab initio* effective core potentials for molecular calculations. Potentials for the transition metal atoms Sc to Hg. *J Chem Phys* 82(1):270–283.

31. Krishnan R, Binkley JS, Seeger R, Pople JA (1980) Self-consistent molecular orbital methods. XX. A basis set for correlated wave functions. *J Chem Phys* 72(1):650–654.

32. Bayly C, Cieplak P, Cornell W, Kollman P (1993) A well-behaved electrostatic potential based method using charge restraints for deriving atomic …. *J Phys Chem*. Available at:

http://pubs.acs.org/cgi-bin/abstract.cgi/jpchax/1993/97/i40/f-pdf/f_j100142a004.pdf?sessid=6006l3.

33. Breneman CM, Wiberg KB (1990) Determining atom-centered monopoles from molecular electrostatic potentials. The need for high sampling density in formamide conformational analysis. *J Comput Chem* 11(3):361–373.

34. Frisch MJ, et al. (2009) Gaussian 09, Revision D.01. *Gaussian Inc*:Wallingford CT.

35. Vallone B, Nienhaus K, Matthes A, Brunori M, Nienhaus GU (2004) The structure of carbonmonoxy neuroglobin reveals a heme-sliding mechanism for control of ligand affinity. *Proc Natl Acad Sci U S A* 101(50):17351–6.

36. Leopold PE, Montal M, Onuchic JN (1992) Protein folding funnels: a kinetic approach to the sequence-structure relationship. *Proc Natl Acad Sci* 89(18):8721–8725.

37. Giordano D, et al. (2012) Biophysical Characterisation of Neuroglobin of the Icefish, a Natural Knockout for Hemoglobin and Myoglobin. Comparison with Human Neuroglobin. *PLoS One* 7(12). doi:10.1371/journal.pone.0044508.

38. Couture M, Burmester T, Hankeln T, Rousseau DL (2001) The heme environment of mouse neuroglobin. Evidence for the presence of two conformations of the heme pocket. *J Biol Chem* 276(39):36377–36382.

39. Ishikawa H, et al. (2007) Neuroglobin dynamics observed with ultrafast 2D-IR vibrational echo spectroscopy. *Proc Natl Acad Sci U S A* 104(41):16116–16121.

40. Sawai H, et al. (2005) Structural characterization of the proximal and distal histidine environment of cytoglobin and neuroglobin. *Biochemistry* 44(40):13257–13265.

41. Howes BD, Helbo S, Fago A, Smulevich G (2012) Insights into the anomalous heme pocket of rainbow trout myoglobin. *J Inorg Biochem* 109:1–8.

42. Morikis D, Champion PM, Springer BA, Sligar SG (1989) Resonance Raman Investigations of Site-Directed Mutants of Myoglobin: Effects of Distal Histidine Replacement. *Biochemistry* 28(11):4791–4800.
